# Incorporating RC-Cornet PLUS and POWERbreathe Medic Plus Device Along With Pulmonary Rehabilitation on Functional Capacity and Quality of Life in Bronchiectasis: A Case Report

**DOI:** 10.7759/cureus.79433

**Published:** 2025-02-21

**Authors:** Amit V Solanke, Lajwanti Lalwani

**Affiliations:** 1 Department of Cardiovascular and Respiratory Physiotherapy, Ravi Nair Physiotherapy College, Datta Meghe Institute of Higher Education and Research, Wardha, IND

**Keywords:** breathlessness, bronchiectasis, chronic cough, pulmonary rehabilitation, rc-cornet plus

## Abstract

This case report discusses the physiotherapy management of an adult patient with chief complaints of breathlessness and difficulty expelling out mucus for the past month, with a history of multiple hospitalizations due to recurrent respiratory infections, who was diagnosed as bronchiectasis. In this study, the patient was treated with a combination of the RC-Cornet PLUS (CEGLA Medizintechnik, Montabaur, Germany), an oscillatory positive expiratory pressure (OPEP) device used for airway clearance, and the POWERbreathe Medic Plus (POWERbreathe, England, UK), an inspiratory muscle training (IMT) device, along with tailored pulmonary rehabilitation. Pulmonary rehabilitation focused on patient education, breathing exercises, airway clearance techniques (ACTs), endurance training, strengthening, and energy conservation techniques. The RC-Cornet PLUS device further complemented these interventions and facilitated effective mucus clearance, while the POWERbreathe Medic Plus device aided in improving respiratory muscle strength and endurance. Through this comprehensive approach, significant improvement in dyspnea was observed from grade 3 to grade 1 on the Modified Medical Research Council (MMRC) scale, improvement in inspiratory muscle strength was observed from 9 cmH2O to 12 cmH2O, there was an increase in functional capacity using a six-minute walk test distance from 60 meters to 190 meters, and improvement in the quality of life was observed on the WHO Quality of Life scale from 50% to 52.5% just after two weeks. The study concludes that adding the RC-Cornet PLUS device and POWERbreathe Medic Plus device to the standard pulmonary rehabilitation protocol improves functional capacity and quality of life in bronchiectasis patients.

## Introduction

Bronchiectasis is a chronic lung illness characterized by persistent and permanent enlargement of the bronchial airways and loss of the function of the mucociliary transport mechanism owing to recurring infection contributing to bacterial invasion and mucus collecting throughout the bronchial tree [1]. The condition affects between one in 1,000 and one in 250,000 persons. The condition is more common in women, and the risk of developing it increases with age [2]. It usually happens with a chronic airway infection, which causes inflammation. The primary clinical symptoms are uncontrollable dyspnea, productive cough, and decreased functional capacity [3]. Bronchiectasis is almost always identified by high-resolution computed tomography (HRCT) scanning. The key diagnostic features are as follows: the bronchus' internal width is greater than its adjacent pulmonary artery, failure of the bronchi to taper, and visibility of bronchi in the outer 1-2 cm of the lung fields [4]. Bronchiectasis is characterized by mild to severe airflow restriction that worsens with time. Cole's "vicious cycle hypothesis" is the best-known model of the development of bronchiectasis. Patients typically have mild to moderate airflow restriction. It was also recently proven that there is a progressive reduction in lung function over time, with loss of forced expiratory volume in one second (FEV1) [5]. An overactive inflammatory response destroys the airways. The involved airways (bronchi) become larger, making them less capable of clearing secretions. These secretions increase the number of germs in the lungs, causing airway obstruction and subsequent deterioration of the airways [6]. Low exercise ability and restricted physical activity are common in patients with chronic respiratory disorders and secretion retention, and these factors are directly related to increased dyspnea, exacerbation, and reduced quality of life [7].

Removing secretions by airway clearance techniques (ACTs) has been advocated in patients with chronic productive cough and mucus blockage on chest computed tomography (CT) and in all patients with cystic fibrosis (CF), despite the lack of scientific data indicating its benefits [8]. The effects of ACTs on secretion clearance, gas-liquid interactions, and viscoelastic properties have been proposed. These effects are based on variations in pulmonary volumes, pressures, expiratory flows, the impact of gravity, or the application of vibratory or compressive forces, depending on the approach used [9]. Airway clearance techniques (ACTs) aim to alleviate symptoms, reduce the frequency of exacerbations, and enhance quality of life. ACTs include instrumental techniques such as positive expiratory pressure devices and high-frequency chest wall oscillation, as well as manual procedures, including manual chest physical therapy, chest percussion, postural drainage, and active cycle breathing techniques [10]. The RC-Cornet PLUS (CEGLA Medizintechnik, Montabaur, Germany) is an oscillatory positive expiratory pressure (OPEP) gadget. It is composed of a semicircular plastic tube. The air exhaled passes through a flexible, latex-free plastic tube that is curved. The latex-free hose makes contact with the top and bottom of the plastic tube as the patient exhales, creating oscillations and positive expiratory pressure in the patient's airway as well as intermittent flow obstruction. There are adjustable settings on the mouthpiece to twist the tube and enlarge the expiratory resistor. The average pressure, amplitude, and frequency may all be altered using this. The Cornet may be used from any posture and is not affected by gravity. The RC-Cornet® has evolved into RC-Cornet® PLUS. Its integrated nebulizer port facilitates more efficient medicine distribution and secretion mobilization [11].

Inspiratory muscle training (IMT) specifically refers to the use of devices that provide resistance during inhalation, forcing the inspiratory muscles to work harder. These devices come in various forms, including handheld devices and specialized equipment. To strengthen the inspiratory muscles, the training typically involves exercises that target the respiratory muscles, such as diaphragmatic breathing exercises, inspiratory muscle resistance training using devices such as inspiratory muscle trainers (IMTs), and exercises to improve overall lung capacity. IMT aims to enhance breathing performance and endurance. It is often used in rehabilitation settings and by athletes looking to improve their respiratory efficiency. IMT is a quantitative resistance technique that works the inspiratory muscles, such as the diaphragm and intercostals, by applying resistance during inspiration. The inspiratory muscles adjust to overcome the "resistance" to provide the effect of IMT. Additionally, inspiratory muscle training can increase endurance and strength and decrease breathing fatigue. It was demonstrated that IMT has additional benefits of improved pulmonary function, respiratory muscle strength and endurance, functional capacity, physical activity, and health-related quality of life (HRQoL), as well as decreased emotional problems and healthcare utilization. Even when used for a short time, IMT showed a greater rehabilitative impact than respiratory exercise alone [12].

## Case presentation

A 38-year-old male farmer visited the outpatient department of respiratory medicine with chief complaints of persistent yellowish productive cough, difficulty in clearing sputum for one month, and breathlessness when performing activities of daily living (ADLs) such as walking, stair climbing, working on the farm, and lifting weights for 15 days, with an associated complaint of generalized weakness since one month. He was admitted to the male respiratory ward, where several investigations were done, such as chest X-ray, HRCT, blood analysis, and sputum analysis, which suggested bronchiectasis. With the same complaints and presentation, he was referred to the physiotherapy department. Thus, physiotherapy sessions started, combining nebulized medications along with inpatient respiratory therapy, which includes patient education, breathing exercises, airway clearance techniques, endurance training, stretching and strengthening exercises, energy conservation techniques, and relaxation techniques that continued for two weeks. After two weeks, the patient was discharged. Unfortunately, after a month, the patient experienced an acute exacerbation of breathlessness and productive cough and was readmitted to the hospital. A subsequent proper assessment, education, and counseling of the patient, as well as his caretaker, were done. A well-structured, progressive, patient-specific protocol was established and implemented, with recent and advanced respiratory devices RC-Cornet PLUS for airway clearance in the acute phase of bronchiectasis exacerbation and POWERbreathe Medic Plus (POWERbreathe, England, UK) for respiratory muscle strengthening in the later post-stabilization phase in adjunct with conventional physiotherapy protocol. The patient had a history of multiple hospitalizations due to recurrent respiratory infections and acute respiratory exacerbations despite adherence to bronchodilators, inhaled corticosteroids, antibiotics, and other asthma medications. Initial evaluation revealed concurrent diagnoses of bronchial asthma along bronchiectasis, posing challenges in symptom control and exacerbation prevention.

Clinical findings

On the day of the assessment, the patient's verbal consent was obtained before the commencement of the detailed physiotherapy assessment. On examination, the body build of the patient was ectomorphic with a stooped posture. The patient was afebrile, with an oxygen saturation of 92% with 6 L of O2 via a nasal prong. Chest examination revealed pectus excavatum deformity, withdrawal, and hollowness of the supraclavicular fossa, with the use of accessory muscles for respiration, reduced chest expansion, shallow breath, and thoracoabdominal breathing pattern.

Diagnostic investigations

**Figure 1:**
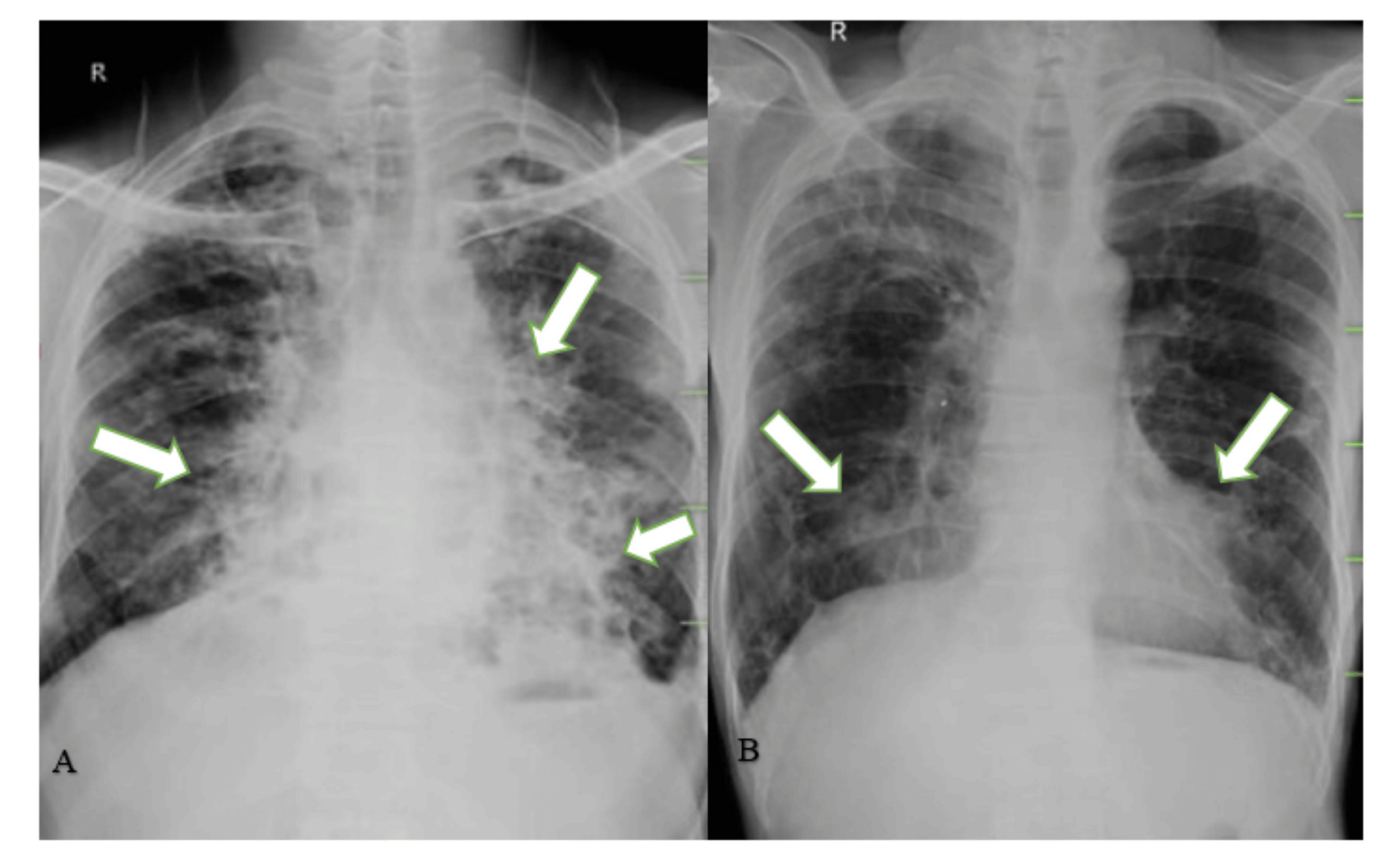
A: Baseline chest X-ray showing marked bronchovascular and hilar markings and the presence of heterogenous opacities bilaterally. B: Post-intervention X-ray after six weeks showing recovery compared to baseline X-ray. A: The right and left side arrows show marked bronchovascular and hilar markings and the presence of heterogenous opacities bilaterally. B: The right and left side arrows signify the markedly reduced bronchovascular and hilar markings.

**Figure 2:**
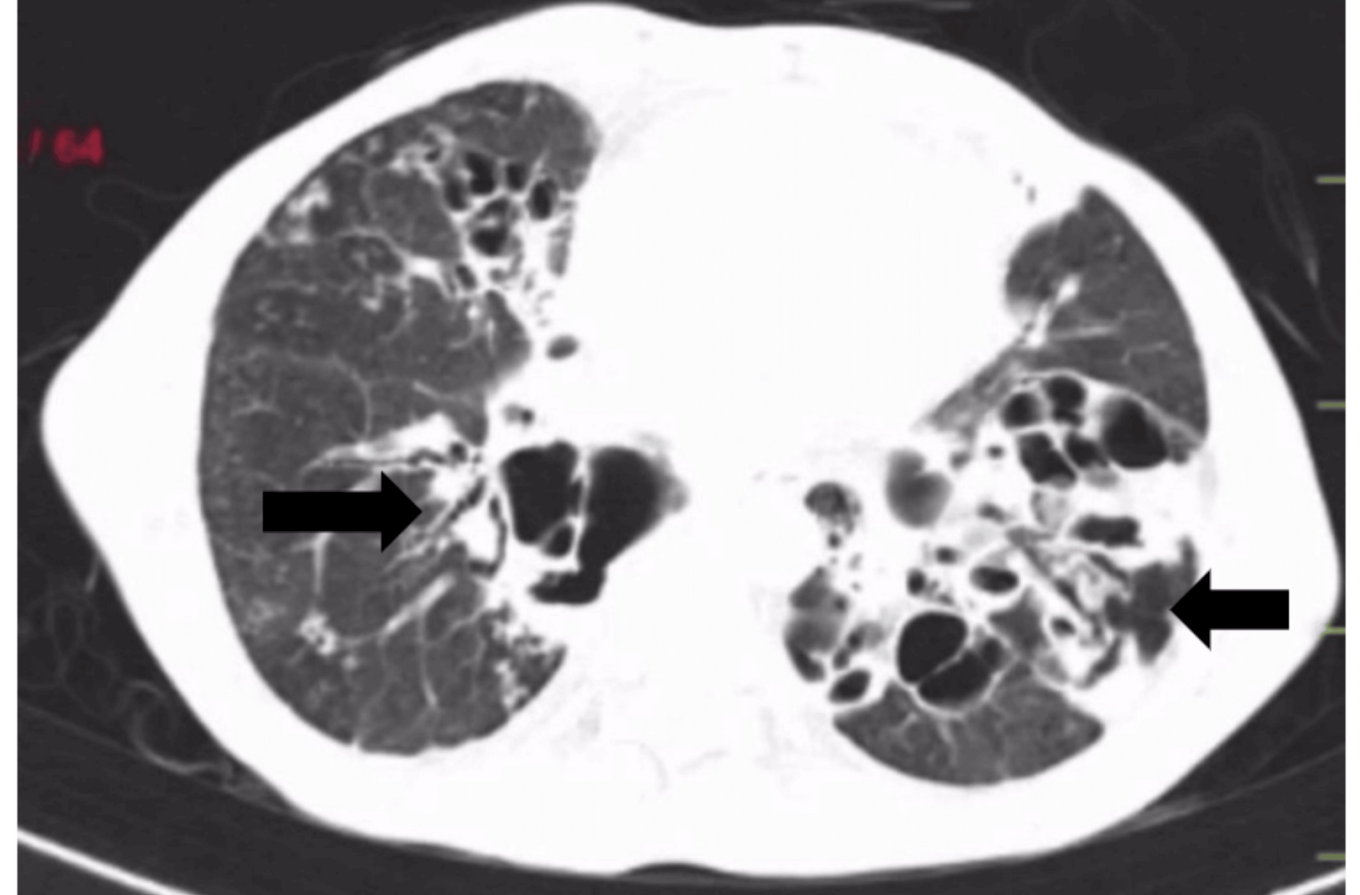
HRCT of the thorax suggesting multiple patchy areas of consolidation and tree-in-bud appearance scattered in bilateral lung fields. Bronchiectatic changes were noted in bilateral lung fields. The right arrow shows the tracheal diverticulum, and the left arrow shows the bronchial diverticulum. HRCT: high-resolution computed tomography

Diagnostic investigations included blood analysis, sputum analysis, pulmonary function test, arterial blood gas analysis, electrocardiogram, chest X-ray (Figure 1A and Figure 1B), and HRCT (Figure 2).

Sputum analysis revealed plenty of degenerated polymorphs, fibrin exudates, debris of cells, and many coccobacilli in the background, suggesting acute suppurative inflammation of the lung parenchyma.

Physiotherapy intervention

An integrated management approach was adopted, as well as targeted intervention for bronchiectasis, emphasizing the role of comprehensive respiratory treatment. The patient's regimen was adjusted to therapies for bronchiectasis, including airway clearance techniques and the use of mucolytic agents. Additionally, the patient was treated using the RC-Cornet PLUS device (OPEP) to enhance airway clearance in the early phase and the POWERbreathe Medic Plus device (threshold IMT) in the later phase of recovery and rehabilitation to improve respiratory muscle strength along with the traditional treatment. The practical outline of the structured, progressive protocol that was implemented in various phases is shown in Table 1.

**Table 1:** Phase-wise management protocol regimen with the goal of intervention, intervention strategy, and dosage along with the progression OPEP: oscillatory positive expiratory pressure, ACBT: active cycle of breathing technique, ADL: activities of daily living, IMT: inspiratory muscle training, EMT: expiratory muscle training, MIP: maximal inspiratory pressure, PIMax: maximum inspiratory pressure, FITT: frequency, intensity, type and time

Goal	Management	Dosage
Week 1 (phase of acute exacerbation)		
Medical stabilization	Medications include antibiotics, bronchodilators, and anti-inflammatory agents	As prescribed by the physician
Oxygen therapy	Administration of supplemental oxygen as needed to maintain adequate oxygen level	According to the needs of the patient
Inpatient respiratory therapy	Patient education, dyspnea relieving positioning, relaxation technique, pursed lip breathing exercise, RC-Cornet PLUS with ACBT	10 minutes thrice daily or whenever needed
Week 2 (post-acute stabilization/assessment of pulmonary rehabilitation suitability and exercise testing/pre-outcome measure assessment)		
To reduce the work of breathing and improve ventilation	Positioning pursed lip breathing exercise	5 repetitions × 3 sets every 4 hours
To improve breathing control	Diaphragmatic breathing exercises	10 repetitions × 1 set every 4 hours
To clear airways and maintain bronchial hygiene	Airway clearance techniques: OPEP device, ACBT	3-5 cycles/day or whenever needed
To reduce fatigue and tiredness and promote relaxation	Energy conservation technique, relaxation therapy (Jacobson's relaxation technique)	10-15 minutes, 2-3 times/day
To improve posture	Posture correction exercises, trapezius and levator stretch, scapular muscles and shoulder external rotator strengthening, pectoral stretching, back extensor stretching, hams, quads, and calf stretching and isometric strengthening; ergonomic advice: during rest and while performing ADL	All the stretching exercises for a 30-second hold with 3 repetitions; the ergonomic advice should be followed while doing fieldwork on the farm (15)
Progressive rehabilitation protocol (reinitiation of pulmonary rehabilitation and exercise prescription)		
To encourage patient	Psychosocial support (emphasizing the importance of ongoing participation)	Before starting the session and whenever needed
To improve respiratory muscle strength	Resisted breathing exercises, respiratory muscle strengthening: IMT using POWERbreathe Medic Plus device, EMT threshold PEP	5 repetitions × 3 sets 5-6 times/week (progressive resistance) (MIP: by PImax, MicroRPM device (Micro Medical/CareFusion, Kent, UK))
To improve endurance	Endurance training and aerobic exercises, including uphill walking, ascending and descending stairs, treadmill walking, and an ergonomic bicycle	According to the FITT principle; frequency: 3-5 days/week, intensity: moderate intensity (i.e., 4-6 on the Borg C-R 10 scale), time: >20 minutes/day of exercise interspersed with intermittent exercise rest periods of lower intensity work or rest, type: interval training
Integration into daily life (education and self-management, promoting independence, regular monitoring, and follow-up) (home exercise program along with lifestyle modifications)		

Endpoints

Endpoints were evaluated at the onset of exacerbation (baseline), the end of exacerbation (day 14), and six weeks post-exacerbation.

The outcome measures used were the Modified Medical Research Council (MMRC) scale, Leicester Cough Questionnaire (LCQ), six-minute walk test (6MWT) distance, and WHO Quality of Life questionnaire. The findings of the outcome measures are shown in Table 2.

**Table 2:** Outcomes at baseline, two weeks, and six weeks MMRC: Modified Medical Research Council, LCQ: Leicester Cough Questionnaire, MIP: maximal inspiratory pressure

Outcome measure	Pre-physiotherapy intervention baseline	Post-physiotherapy intervention after 2 weeks	Follow-up after 6 weeks
MMRC scale	Grade 3	Grade 1	Grade 1
LCQ	37/133	69/133	73/133
6-minute walk test	60 meters	190 meters	280 meters
WHO Quality of Life questionnaire	50%	52.5%	57%
MIP	9 cmH2O	12 cmH2O	18 cmH2O

## Discussion

A 38-year-old male farmer by occupation came with a chief complaint of persistent yellowish productive cough, difficulty in sputum clearance for one month, and breathlessness in performing ADLs such as walking, stair climbing, working on the farm, and lifting weights for 15 days, with associated complaints of generalized weakness since one month. He is diagnosed with bronchiectasis along with asthma. A poorer prognosis, a higher possibility of exacerbations, and severe pulmonary insufficiency are all due to this underlying disease. From a clinical perspective, to lower future risks, this patient subgroup may require specialized treatment aimed at clearing their airways in addition to the standard protocols for asthma patients [13].

A comprehensive physiotherapy regimen including a phase- and symptom-guided protocol along with standard pulmonary rehabilitation was administered to address the patient's symptoms. The RC-Cornet PLUS device, combined with the active cycle of breathing technique, was employed to facilitate secretion mobilization, reduce dyspnea, and improve lung aeration and breathing efficiency. The patient exhibited subjective ease in facilitating secretion clearance and enhanced satisfaction after the use of the device. Additionally, mobility exercises were used to maintain and enhance physical function. It is, therefore, deserving of therapeutic promotion and application to combine respiratory rehabilitation training with limb exercise rehabilitation to enhance the sputum clearance rate, lung function, and quality of life of patients with bronchiectasis [14].

Further, to improve the patient's aerobic capacity, lung capacity, and endurance, an enhanced pulmonary rehabilitation program was implemented. As a result of this comprehensive approach, the patient experienced significant improvements in dyspnea, functional capacity, and quality of life. These advancements enabled the patient to resume daily activities and return to work on the farm, showcasing the effectiveness of the physiotherapy interventions in boosting respiratory efficiency and overall physical functioning. This highlights the critical role of cardiopulmonary physiotherapy in maintaining and enhancing patient function, particularly in supporting return to work [15].

The effectiveness of OPEP devices in managing bronchiectasis is well-established in the literature and is widely utilized across various respiratory conditions for airway clearance. A systematic review by Lee et al. was conducted examining the effects of OPEP therapy in adults with stable non-cystic fibrosis bronchiectasis, highlighting its effectiveness in enhancing sputum expectoration and improving disease-specific quality of life [10]. Compared to other ACTs, even the short-term use of OPEP enhances lung function, gas exchange, and symptom relief. However, its impact on reducing exacerbation rates remains unclear. The review consolidates evidence from seven studies, showing that OPEP therapy is a viable option in managing stable non-cystic fibrosis bronchiectasis, with higher preference and efficacy than other established ACTs [16]. The present study incorporated the RC-Cornet PLUS, a more recent and advanced OPEP device that offers multiple frequency and resistance settings. It can oscillate at low flow rates, around 2 cmH2O, allowing patients to adjust the mouthpiece for optimal pressure and airflow, making it particularly suitable for patients with low tidal volumes. Also, great compatibility and adherence were documented for the RC-Cornet PLUS compared to other devices and other methods for airway clearance [17]. RC-Cornet PLUS, when combined with the active cycle of breathing technique, also showed good results for the removal of secretions in the acute exacerbation phase. This helps reduce dyspnea as the lung fields are clear enough for air to enter the lung fields.

To develop patients' overall endurance and respiratory efficiency, we implemented an enhanced pulmonary rehabilitation program. Post-treatment assessments revealed significant positive changes, including reduced breathlessness and increased functional capacity. The WHO Quality of Life questionnaire showed a statistically significant improvement in physical, social, and psychological domains from pre-treatment to post-treatment, with further gains observed during follow-up. These results underscore the value of early, comprehensive cardiopulmonary physiotherapy rehabilitation in promoting functional independence and facilitating successful return to work.

A systematic review and meta-analysis conducted by Figueiredo et al. on inspiratory muscle training (IMT) in patients with COPD concluded that isolated IMT significantly improves inspiratory muscle strength, functional capacity, and pulmonary function [18]. However, it does not substantially alter dyspnea or quality of life. These findings suggest that isolated IMT can be effectively used as an adjunctive intervention in COPD management. No favorable results were found for dyspnea and QOL, but in the present study, these parameters significantly improved. This may have been due to the use of the RC-Cornet PLUS device in our intervention for airway clearance, which must have lessened dyspnea and QOL.

According to the literature, devices are proven to be more effective for early muscle training than conventional treatment methods. A randomized controlled trial by Ozalp et al. sought to investigate the effect of high-intensity IMT (H-IMT) on bronchiectasis patients' ability to exercise [19]. Forty-five patients with non-cystic fibrosis bronchiectasis participated in the trial and were divided into two groups: the H-IMT group and the control group. Over eight weeks, the H-IMT group trained at 70% of their maximal inspiratory pressure (MIP), while the control group trained at 10% of MIP. The H-IMT group showed significant improvements in MIP, maximal expiratory pressure (MEP), shuttle walk distance, respiratory muscle endurance, fatigue reduction, and social aspects of quality of life. This study demonstrated that IMT increases the velocity of inspiratory muscle contraction and enhances both inspiratory and expiratory times, as measured by pressure-time units and constant threshold load. These observations correspond to the findings of the present case report study, which showed a greater improvement in dyspnea in only two weeks and 6MWT distance in six weeks of a patient-tailored intervention regimen.

Ceyhan et al. conducted a study on the predictive role of psychological status and disease severity indexes on quality of life among patients with non-CF bronchiectasis [20]. The results emphasize the importance of mental health and disease severity as significant determinants of life quality in patients, particularly female patients with non-cystic fibrosis bronchiectasis. Also, McCreery et al. evaluated the effects of an eight-week high-intensity inspiratory muscle training program using the PrO2Fit device in adults with bronchiectasis [21]. The study involved 18 participants who underwent three weekly sessions at 80% sustained maximal inspiratory pressure (SMIP). The results indicated significant improvements in lung function, respiratory muscle strength, endurance, exercise capacity, and physical activity levels.

Additionally, participants reported enhanced perceived competence, motivation, and adherence to the program, alongside improved physical ability. The IMT program positively impacted inspiratory muscle strength, endurance, and participant's sense of autonomy and competence. This significant improvement must be because of the perceived competence, motivation, and adherence to the intervention, which is much higher on using devices and progressive monitored programs.

The present study highlights the importance of utilizing a multifaceted treatment strategy, emphasizing the role of advancement in existing literature for respiratory devices and patient-tailored rehabilitation programs in optimizing quality of life.

## Conclusions

The study concludes that incorporating RC-Cornet PLUS and POWERbreathe Medic Plus devices in the standard pulmonary rehabilitation protocol improves functional capacity and quality of life in bronchiectasis patients. Therefore, recent and advanced respiratory devices for airway clearance and inspiratory muscle strengthening devices should be considered and incorporated as an adjunct to the standard pulmonary rehabilitation regimen for bronchiectasis patients.
